# Activation of the Nrf2/HO-1 pathway restores N-acetylcysteine-induced impairment of the hypothalamus-pituitary-adrenal axis negative feedback by up-regulating GRα expression and down-regulating GRβ expression into pituitary glands

**DOI:** 10.3389/fendo.2025.1500630

**Published:** 2025-01-27

**Authors:** Amanda Silva Chaves, Raíssa Duarte Ventura, Maria Florencia Pacini, Nathalia Santos Magalhães, Patrícia Machado Rodrigues e Silva, Marco Aurélio Martins, Ana Rosa Pérez, Vinicius Frias Carvalho

**Affiliations:** ^1^ Laboratory of Inflammation, Center for Research, Innovation, and Surveillance in Covid-19 and Health Emergencies, Oswaldo Cruz Institute, Oswaldo Cruz Foundation, Rio de Janeiro, Brazil; ^2^ Institute of Clinical and Experimental Immunology (IDICER-CONICET UNR), Rosario, Argentina; ^3^ National Institute of Science and Technology on Neuroimmunomodulation (INCT-NIM), Oswaldo Cruz Institute, Oswaldo Cruz Foundation, Rio de Janeiro, Brazil; ^4^ Rio de Janeiro Research Network on Neuroinflammation (RENEURIN), Oswaldo Cruz Institute, Oswaldo Cruz Foundation, Rio de Janeiro, Brazil; ^5^ INOVA-IOC Network on Neuroimmunomodulation, Oswaldo Cruz Institute (IOC), Oswaldo Cruz Foundation, Rio de Janeiro, Brazil

**Keywords:** antioxidants, glucocorticoid, GR isoforms, HPA axis, Nrf-2

## Abstract

We previously showed that antioxidants induced an impairment of negative feedback of the hypothalamus-pituitary-adrenal (HPA) axis in rats, in parallel to a down-regulation of the glucocorticoid receptor (GR) and nuclear factor erythroid 2-related factor 2 (Nrf2) expression in the pituitary gland. This study evaluated the role of the Nrf2-heme-oxygenase-1 (HO-1) pathway on the impairment of the negative feedback of the HPA axis induced by N-acetylcysteine (NAC). Male Swiss-Webster mice were orally supplemented with NAC for 5 consecutive days. The Nrf2-HO-1 pathway activator cobalt protoporphyrin IX (CoPPIX) was injected intraperitoneally on days 2 and 5 after the starting of NAC supplementation. NAC reduced the expression of Nrf2 in the pituitary of mice. Furthermore, NAC induced adrenal enlargement and hypercorticoidism, along with a decrease in the GRα expression and an increase of GRβ expression in the pituitary gland. Treatment with CoPPIX reduced adrenal enlargement, systemic corticosterone levels, and GRβ expression in the pituitary gland of mice supplemented with NAC, besides increasing the expression of GRα. CoPPIX treatment also restored the failure in the negative feedback of the HPA axis induced by NAC. In conclusion, these findings showed that NAC reduced the Nrf2-HO-1 pathway activation in the pituitary gland, in a mechanism probably related to a local downregulation of GRα and an up-regulation of GRβ, leading to a failure of negative feedback of the HPA axis and consequently to the hyperactivity of this neuroendocrine axis.

## 1 Introduction

The hypothalamus-pituitary-adrenal (HPA) axis is a neuroendocrine system regulated by the circadian cycle and stress (1). Under normal conditions, glucocorticoids, the final product of the HPA axis, are produced through the activation of the adrenocorticotropic hormone (ACTH)-melanocortin receptor 2 (MC2R) signaling pathway in the adrenal glands (2). Chronic activation of the HPA axis can have damaging effects on immune, cardiovascular, metabolic, and neural functions, increasing the risk of immune system dysfunction, mood disorders, and metabolic and cardiovascular diseases (3). To prevent these deleterious effects of chronic hypercortisolism, the HPA axis function is controlled by a glucocorticoid-dependent negative feedback system that is essential for ending the stress response. In corticotroph cells, the activation of glucocorticoid receptor (GR)-α isoform by glucocorticoids is crucial for the negative feedback of the HPA axis (4).

Reactive oxygen species (ROS), such as superoxide, hydroxyl radicals, and hydrogen peroxide, are produced by oxygen reduction (5). As the overproduction of ROS is toxic to cells, its synthesis is finely controlled by endogenous antioxidant enzymes (6). ROS are known to act as signaling molecules in physiological processes, activating a defensive response, including nuclear factor erythroid 2-related factor 2 (Nrf2)/heme oxygenase (HO)-1 pathway, which appears to protect the organism from subsequent higher stresses (7–9). Furthermore, the loss of Nrf2 activity in mouse fibroblasts, hepatocytes, and liver cells disrupts local circadian rhythms (10). Although the disruption of local circadian rhythms can increase pituitary gland activation (11, 12), the effect of Nrf2 on the negative feedback of the HPA axis is unclear.

In previous works, we demonstrated that oral supplementation with antioxidants induces hyperactivity of the HPA axis in mice and rats (13, 14). The antioxidant-induced hyperactivation of this axis was related to overexpression of MC2R and steroidogenic machinery in the adrenal gland, along with a down-regulation of GR in the pituitary gland, leading to a failure of the negative feedback mechanism of the HPA axis. We also showed that N-acetylcysteine (NAC) reduced the expression of Nrf2 and HO-1 in the pituitary gland of rats (13). In this study, we evaluated the effect of Nrf2/HO-1 pathway activator cobalt protoporphyrin IX (CoPPIX) (15, 16) on the NAC-induced hypercortisolism in healthy mice.

## 2 Materials and methods

### 2.1 Animals

Following the guidelines of the Committee on Use of Laboratory Animals of Oswaldo Cruz Institute (CEUA-IOC/Fiocruz, license L-027/2016 and L-004/2024), male Swiss-Webster mice obtained from Science and Technology in Biomodels Institute of Fiocruz were used. Mice aged between 4 and 6 weeks were housed in groups of five in temperature-, humidity-, and light-controlled (12 h light: 12 h darkness cycle) colony room. Mice were given access *ad libitum* to food and water.

### 2.2 Antioxidant supplementation and treatment

**Figure 1:**
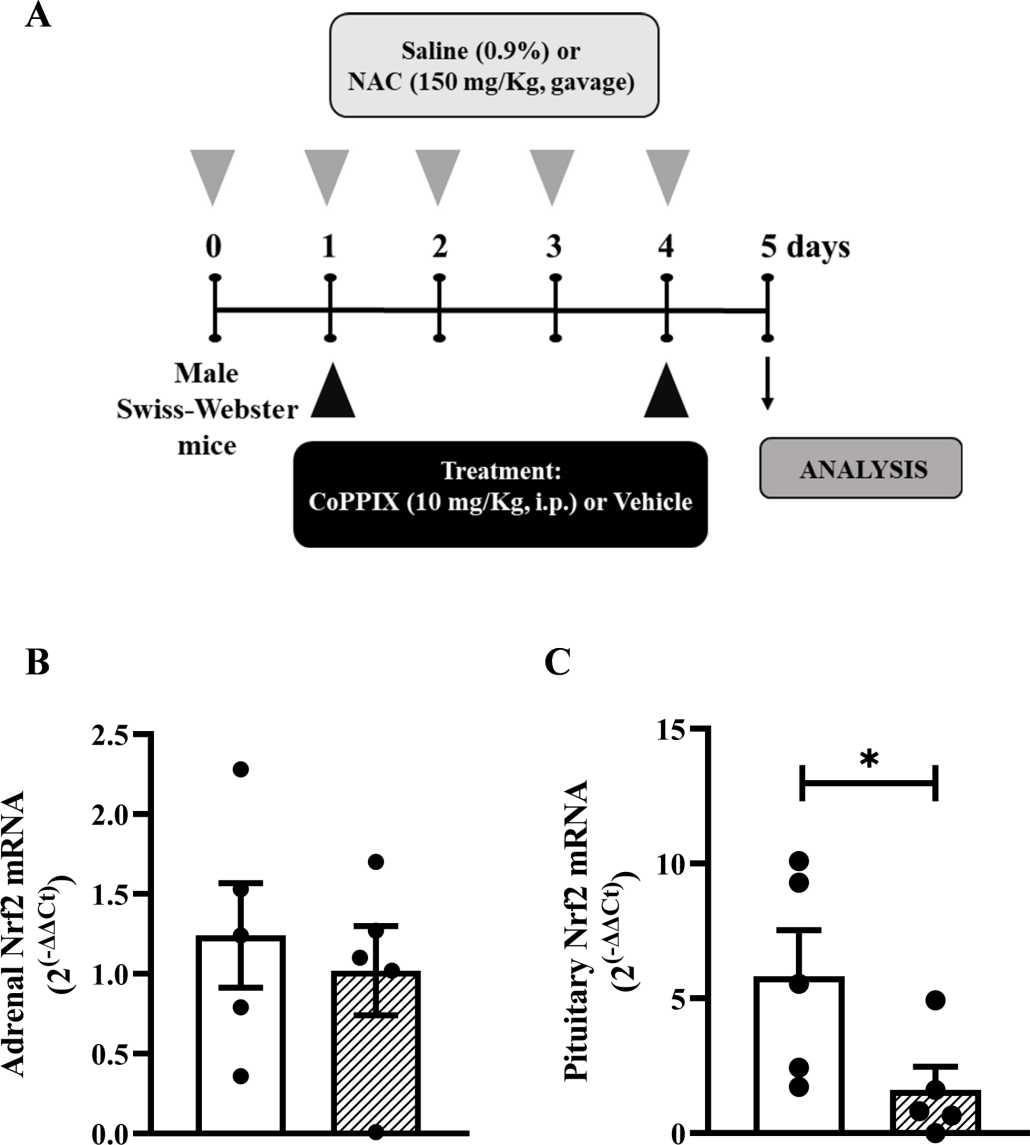
CoPPIX increases the under-expression of Nrf2 and HO-1 in the pituitary glands of mice supplemented with NAC. **(A)** *In vivo* protocol. Mice were supplemented with NAC (150 mg/Kg, gavage) daily for 5 consecutive days and treated with CoPPIX (10 mg/Kg, i.p.) on days 2 and 5 after the starting of oral supplementation with the antioxidant. Non-supplemented animals received an equal amount of vehicles (NaOH 0.1N and HCl 1N, pH = 7.4, i.p.), and analyses were performed 24 h after the last supplementation with NAC and/or treatment with CoPPIX. **(B, C)** Nrf2 gene expression in adrenal and pituitary glands of mice, measured by qPCR, respectively. Data are expressed as the mean ± SEM. *P < 0.05. CoPPIX, Cobalt protoporphyrin IX; HO-1, Heme-oxygenase-1; NAC, N-acetylcysteine; Nrf2, Nuclear factor erythroid 2-related factor 2.

The mice were supplemented with antioxidant N-acetyl cysteine (NAC) (150 mg/kg body weight) (Sigma Chemical Co., Saint Louis, MO, USA) (17–19) by gavage once a day, during five consecutive days. Control mice received an equal volume of vehicles (sterile saline 0.9%). The mice were treated concomitantly with Co (III) Protoporphyrin IX chloride (Nrf2-HO-1 pathway activator – CoPPIX) (10 mg/kg body weight, i.p.) (Co654-9, Frontier Scientific, Inc., Logan, UT, USA) (14) on days 2 and 5 after the start of NAC supplementation (**Figure 1A**). CoPPIX was diluted with NaOH 0.1N (10% final volume) (Merck, Rio de Janeiro, Brazil), and then, HCl 1N (Merck) to obtain a solution with pH 7.4. Untreated mice received an equal volume of vehicles. To analyze corticoid-induced negative feedback sensitivity, a group of animals received dexamethasone (20 µg/kg body weight, s.c.) (Sigma Chemical Co.) 1h before the euthanasia. Control mice were treated s.c. with sterile saline 0.9%. All analyses were performed 24 hours after the last supplementation with NAC. All solutions were freshly prepared immediately before use. To analyze whether the effect of NAC is sustainable after the interruption of the supplementation, we administered NAC by gavage once a day, during five consecutive days, and analyzed plasma corticosterone levels 24h and 15 days after the last supplementation with NAC.

### 2.3 Evaluation of mRNA expression of GRα, GRβ, and Nfr-2 by real time PCR

Total RNA was isolated from adrenal and pituitary glands using TRI Reagent^®^ and followed reverse-transcribed to cDNA using the RevertAid Reverse Transcriptase (Thermo Fisher Scientific, Waltham, USA). Real-time PCR was performed with the StepOnePlus Real-Time PCR System (Applied Biosystems, Foster City, USA) using a Mix (5x HOT FIREPol^®^ EvaGreen^®^ qPCR Mix Plus with ROX; Solis BioDyne, Estonia) according to the manufacturer’s instructions. The amplification program included an initial activation step at 95°C for 15min, followed by denaturation at 95°C for 15s, annealing between 59°-62°C and finally elongation at 72°C for 20s, for 40 cycles. Fluorescence was measured after each extension step, and the specificity of amplification was evaluated by melting curve analysis. The housekeeping gene GADPH was used as a control to normalize RNA samples. Relative gene expression levels were calculated using the ΔΔC_T_ method (20). Amplification efficiencies were identical or similar between genes of interest and controls. Primers were designed in our laboratory and purchased from Eurofins Genomics Scientific, Inc. (Louisville, USA) or Invitrogen Thermo Fisher Scientific, Inc. (Carlsbad, USA). The primer used were: i) GAPDH - forward: AGCAATGCATCCTGCACCACCA; reverse: ATGCCAGTGAGCTTCCCGTTCA; ii) GRα - forward: AAAGAGCTAGGAAAAGCCATTGTC; reverse: TCAGCTAACATCTCTGGGAATTCA; iii) GRβ - forward: AAAGAGCTAGGAAAAGCCATTGTC; reverse: CTGTCTTTGGGCTTTTGAGATAGG; iv) Nrf2 - forward: TAGATGACCATGAGTCGCTTG; reverse: GCCAAACTTGCTCCATGTCC.

### 2.4 Western blot analysis

The pituitary glands were homogenized in RIPA buffer containing protease and phosphatase inhibitor cocktails, and then, the protein content was quantified by the BCA method (21). 60 μg total protein/lane was resolved on 12% sodium dodecyl sulfate-polyacrylamide gel electrophoresis, for evaluation of GR protein expression, and afterward electrotransferred through a semi-dry transfer apparatus (Trans-Blot SD; Bio-Rad, Hercules, CA, USA) to a nitrocellulose membrane. Subsequently, the membrane was blocked with a solution containing Tris-buffered saline, 5% bovine serum albumin, and 0.1% Tween 20, pH 7.4 (TBST), for 90 minutes at room temperature. Then, the membrane was incubated with a primary antibody dissolved in the blocking solution overnight at 4°C. Primary antibody against anti-GR (1:200; Santa Cruz Biotechnology) was used. The housekeeping anti-β-actin (1:1000; Santa Cruz Biotechnology) was used as the standard. Afterward, the membrane was incubated with an HRP conjugated secondary antibody polyclonal anti-rabbit IgG HRP (1:10.000, Invitrogen ThermoFisher Scientific, MA, USA), or monoclonal anti-mouse IgGs HRP (1:1000, R&D System, Minneapolis, MN, USA) for 60 minutes at room temperature, and the immunocomplexes were visualized by using a ChemiDoc MP Imaging System 6.0.1 (Bio-Rad Laboratories, Inc, Hercules, CA, USA). Then, the band density measurements were analyzed by Image Lab software version 6.1.0 (Bio-Rad Laboratories, Inc). The description of all the antibodies used is in **Supplementary Table S1**.

### 2.5 Hormone quantification

After euthanasia (ketamine 140 mg/Kg and xylazine 20 mg/Kg i.p.) of mice, during nadir (08:00h) of the circadian rhythm (21). Blood was immediately collected from the abdominal aorta with heparinized (40 U/ml) saline, centrifuged for 20 min at 1000 x g, and stored at -20°C until use. The ELISA kit detected plasma corticosterone levels following the manufacturer’s guidelines (Cayman Chemical, 501320, Cedarlane Labs, Canada).

### 2.6 Statistical analysis

The data are reported as the mean ± standard error of the mean (SEM). Data distribution was assessed by Kolmogorov-Smirnov. Data with normal distribution was statistically analyzed by one-way ANOVA followed by Tukey’s multiple comparison *post-hoc* test. Data without normal distribution was evaluated by Kruskall-Wallis followed by the U-Mann Whitney test. All statistical analysis was performed with GraphPad Prism 8 software. Probability values (p) of 0.05 or less were considered significant. Determining if the frequency distribution of a given data set follows a normal distribution or not is among the first steps of data analysis.

## 3 Results

### 3.1 CoPPIX restores NAC-induced under-expression of Nrf-2 in the pituitary gland of mice

Supplementation with NAC (**Figure 1A**) did not alter the mRNA expression of Nrf2 in adrenal glands compared to non-supplemented mice (**Figures 1B**); however, it reduced the mRNA expression of Nrf2 in the pituitary glands (**Figures 1C**).

### 3.2 CoPPIX reduces adrenal enlargement and hypercorticoidism induced by NAC supplementation

**Figure 2:**
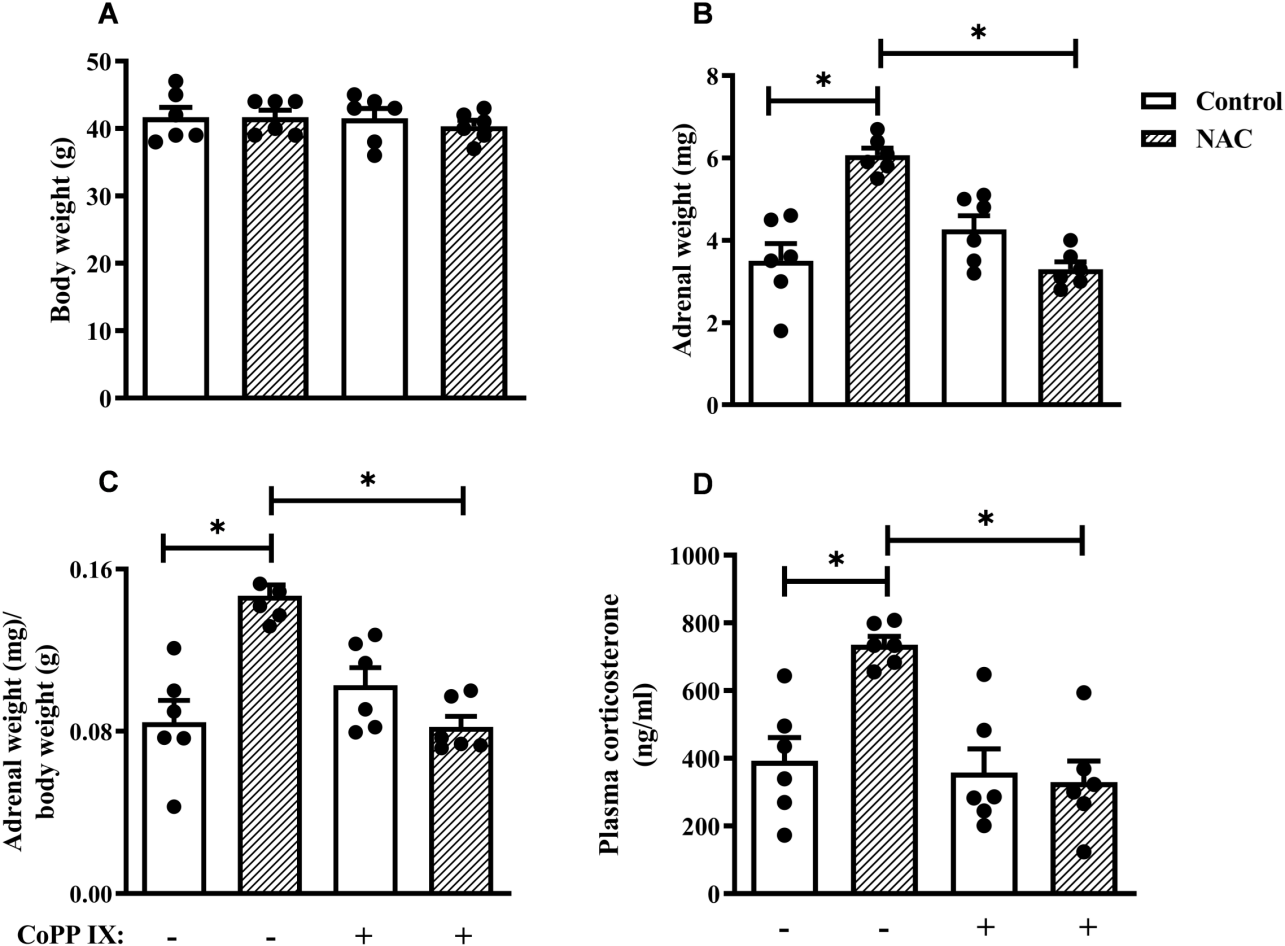
CoPPIX reduces adrenal enlargement and plasma corticosterone levels observed in mice supplemented with NAC. Mice were supplemented with NAC (150 mg/Kg, gavage) daily for 5 consecutive days and treated with CoPPIX (10 mg/Kg, i.p.) on days 2 and 5 after the starting of oral supplementation with the antioxidant. Non-supplemented animals received an equal amount of vehicle (NaOH 0.1N and HCl 1N, pH = 7.4, i.p.), and analyses were performed 24 h after the last supplementation with NAC and/or treatment CoPPIX. **(A)** Body weight of mice. **(B)** Adrenal weight of mice. **(C)** The ratio between adrenal and body weight. **(D)** Plasma quantification of corticosterone levels. Data are expressed as the mean ± SEM. *P < 0.05. CoPPIX, Cobalt protoporphyrin IX; NAC, N-acetylcysteine.

NAC supplementation did not affect the overall body weight of mice (**Figure 2A**), but induced adrenal enlargement, which was evidenced by an increase in the adrenal weight and the adrenal-to-body weight ratio (**Figures 2B, C**, respectively) compared to non-supplemented mice. However, the treatment with CoPPIX prevented the adrenal enlargement observed in NAC-supplemented mice without modifying the body weight (**Figures 2A-C**). CoPPIX did not alter these readouts in non-supplemented mice. In addition, NAC increased circulating corticosterone levels compared to non-supplemented mice (**Figure 2D**). Remarkably, this change was sensitive to CoPPIX treatment, as evidenced by conditions in which the CoPPIX did not alter this parameter in non-supplemented mice (**Figure 2D**).

### 3.3 CoPPIX restored NAC-induced failure in the negative feedback of the HPA axis by increasing the expression of GRα in the pituitary gland of mice

**Figure 3:**
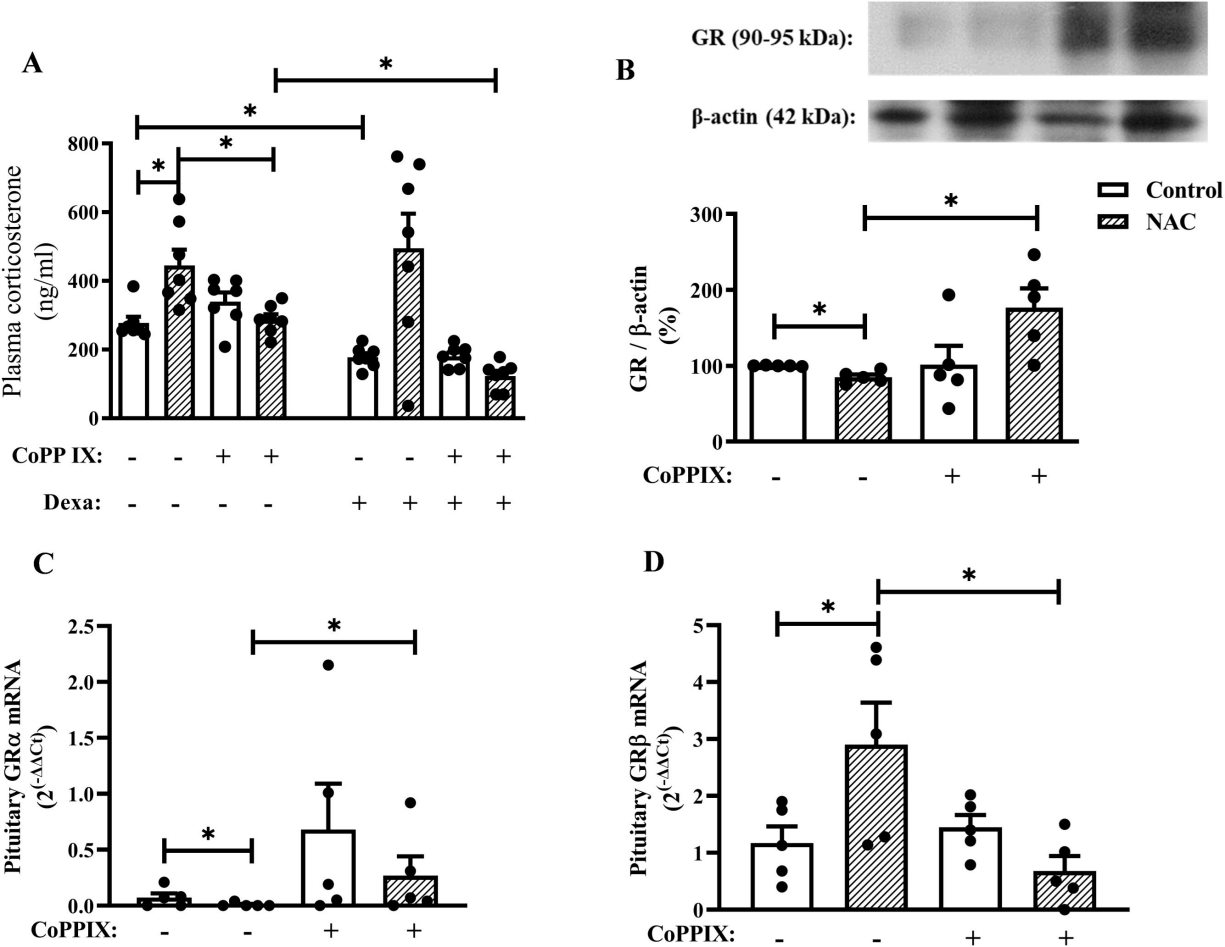
CoPPIX restores the impairment of negative feedback of the HPA axis in mice supplemented with NAC by restoring the imbalance in the GR isoforms in the pituitary gland. Mice were supplemented with NAC (150 mg/Kg, gavage) daily for 5 consecutive days and treated with CoPPIX (10 mg/Kg, i.p.) on days 2 and 5 after the starting of oral supplementation with the antioxidant. Non-supplemented animals received an equal amount of vehicle (NaOH 0.1N and HCl 1N, pH = 7.4, i.p.), and analyses were performed 24 h after the last supplementation with NAC and/or treatment CoPPIX. Some groups of mice were injected with dexamethasone (20 µg/Kg, s.c.) or vehicle (saline 0.9%, s.c.) 1h before de euthanasia. **(A)** Quantification of plasma corticosterone levels after dexamethasone suppression test *in vivo*. **(B)** Expression of total GR in pituitary glands was determined by western blot. The data were normalized to β-actin and represented as the ratio between the expressions of GR: β-actin relative to the control. **(C, D)** GRα and GRβ gene expression in pituitary glands of mice measured by qPCR, respectively. Data are expressed as the mean ± SEM. *P < 0.05. CoPPIX, Cobalt protoporphyrin IX; Dexa, Dexamethasone; GR, glucocorticoid receptor; NAC, N-acetylcysteine.

Dexamethasone reduced plasma corticosterone levels in non-supplemented mice, however; it failed to inhibit this readout in NAC-supplemented mice, indicating an impairment in the negative feedback of the HPA axis due to NAC supplementation in mice. Notably, CoPPIX restored dexamethasone’s ability to reduce plasma corticosterone levels in NAC-supplemented mice, while it did not affect this parameter in non-supplemented mice (**Figure 3A**). We also demonstrated that NAC decreased GR protein expression in the pituitary gland of mice, which was sensitive to CoPPIX treatment (**Supplementary Figure S1**). Furthermore, CoPPIX did not modify this readout in non-supplemented mice (**Figure 3B**). In addition, NAC significantly reduced GRα expression (**Figure 3C**) and increased GRβ expression in the pituitary gland of mice (**Figure 3D**). Treatment with CoPPIX significantly increased GRα expression and decreased GRβ expression in the pituitary gland of mice supplemented with NAC (**Figures 3C, D**, respectively). CoPPIX treatment did not significantly alter GRα and GRβ in non-supplemented mice.

### 3.4 NAC increases plasma corticosterone levels in mice even after discontinuing supplementation for 15 days

**Figure 4:**
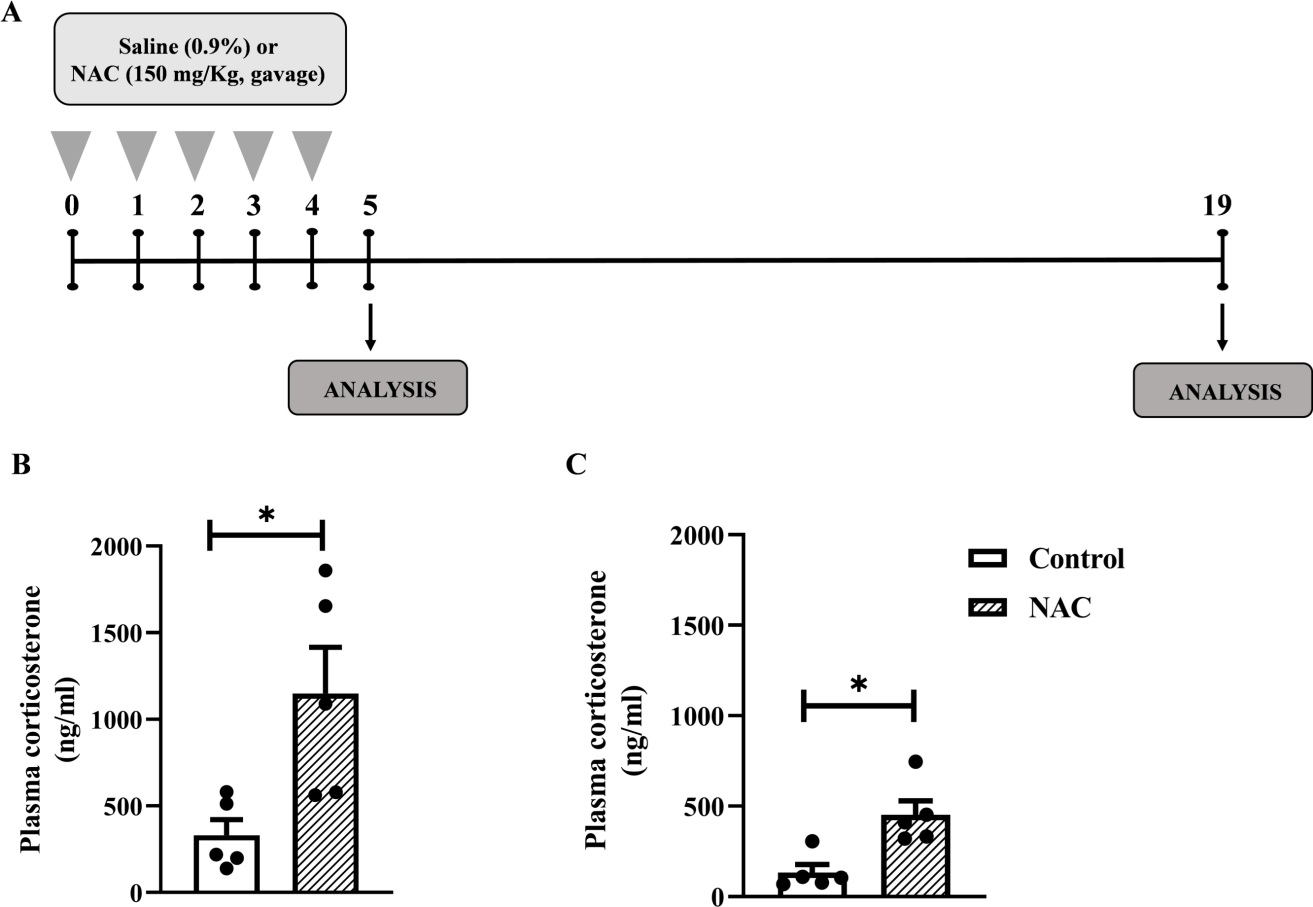
NAC increases plasma corticosterone levels in mice even after discontinuing supplementation for 15 days. **(A)** *In vivo* protocol. Mice were supplemented with NAC (150 mg/Kg, gavage) daily for 5 consecutive days. Non-supplemented animals received an equal amount of vehicles (NaOH 0.1N and HCl 1N, pH = 7.4, i.p **(B)** Quantification of plasma corticosterone levels 24 h after the last supplementation with NAC. **(C)** Quantification of plasma corticosterone 15 days after the last NAC supplementation. Data are expressed as the mean ± SEM. *P < 0.05. NAC, N-acetylcysteine.

We observed that dietary supplementation with NAC (**Figure 4A**) significantly increased plasma corticosterone levels in mice 24h (**Figure 4B**) as well as 15 days (**Figure 4C**) after the last administration of the antioxidant with the same magnitude of the response (3.5-fold and 3.4-fold, respectively).

## 4 Discussion

This study investigated the effect of Nrf2/HO-1 pathway activator CoPPIX on NAC-induced impairment of HPA axis negative feedback in healthy mice. We found that CoPPIX treatment increased the expression of Nrf2 in the pituitary gland of NAC-supplemented mice. In addition, CoPPIX treatment inhibited NAC-induced hypercorticoidism in healthy mice, parallel to a reduction in adrenal enlargement. The decrease in the corticosterone production in the adrenal glands of NAC-supplemented mice treated with CoPPIX was correlated with an improvement in the negative feedback of the HPA axis, as well as up-regulation of GRα and downregulation of GRβ expression in the pituitary gland. Our findings suggest that the impairment of the negative feedback of the HPA axis induced by NAC supplementation is probably due to an imbalance in GR isoform expression in the pituitary gland, caused by local downregulation of the Nrf2-HO-1 pathway.

Currently, many people incorporate antioxidants into their daily regimen as a proactive measure to combat the effects of aging, including the development of age-related diseases (22, 23). Nevertheless, numerous clinical trials evaluating the efficacy and safety of antioxidant supplementation have failed to show beneficial readouts and, in some cases, have suggested an increase in overall mortality rates (24–26). Previously, we showed that supplementation with two different antioxidants, NAC and vitamin E, increased plasma corticosterone levels in mice and rats (13). Additionally, NAC was shown to abolish the ability of exogenous glucocorticoids to perform negative feedback on the HPA axis in rats, alongside a decrease in the expression of GR, Nrf2, and HO-1 in the pituitary gland (13). However, the causal effect of the downregulation of the Nrf2-HO-1 pathway on NAC-induced impairment of the HPA axis negative feedback remains unknown. Therefore, we hypothesized that activating the Nrf2-HO-1 pathway by CoPPIX in the pituitary of NAC-supplemented mice could improve the HPA axis negative feedback failure observed in these animals.

Initially, we confirmed our previous findings in rats and demonstrated that NAC supplementation also reduced the expression of Nrf2 in the pituitary gland of healthy mice, even with a shorter supplementation period. Our data agree with the literature that showed that treatment with NAC reduced the expression of Nrf2 and HO-1 in non-stimulated human umbilical vein cells (HUVECs) and C2C12 myotube cells and AuNP-induced up-regulation of Nrf2-HO-1 pathway in human vascular endothelial cells (27–29).

To evaluate whether activation of the Nrf2-HO-1 pathway in the pituitary of NAC-supplemented mice could improve the negative feedback of the HPA axis, we first evaluated if CoPPIX treatment could reduce the hypercorticoidism observed in these animals. First, we confirmed that supplementation with NAC increased the plasma corticosterone levels in mice. Although antioxidant treatment decreases corticosterone levels in several models of diseases, including brain oxidative stress induced by lipopolysaccharide and streptozotocin-induced diabetes in rats (30, 31), we showed that physiological ROS act as messenger molecules in pituitary cells of healthy mice and are vital to their homeostasis maintenance. Furthermore, we showed that CoPPIX treatment inhibited both NAC-induced adrenal enlargement and hypercorticoidism in mice. These findings suggest that reduced activation of the Nrf2-HO-1 signaling pathway in the pituitary is involved in the hyperactivity of the HPA axis induced by NAC supplementation. While we believe that CoPPIX does not act directly on the adrenal glands – since NAC supplementation did not alter Nrf2 mRNA expression in the adrenals – we cannot entirely rule out the possibility that CoPPIX restored NAC-induced a reduction in the MC2R expression in the adrenal gland and, consequently, the adrenal insensitivity to ACTH stimulation. In fact, the downregulation of HO-1 expression in adrenocortical cells has been shown to increase ACTH-induced progesterone steroidogenesis *in vitro* (32), indicating that the Nrf2-HO-1 pathway plays a crucial role in adrenal steroidogenesis.

Since CoPPIX inhibited NAC-induced hypercorticoidism and restored the expression of Nrf2 and HO-1 in the pituitary gland of mice, we further evaluated the effect of Nrf2-HO-1pathway activation on the functioning of the negative feedback of the HPA axis. For this, we assessed the sensitivity of the HPA axis to negative feedback induced by the synthetic exogenous glucocorticoid dexamethasone in NAC-supplemented mice treated or not with CoPPIX. Dexamethasone reduced circulating corticosterone levels in non-supplemented mice, but did not affect plasma glucocorticoid levels in NAC-supplemented mice, indicating that NAC impairs the negative feedback of the HPA axis. Interestingly, CoPPIX treatment significantly reduced plasma corticosterone levels in dexamethasone-treated and NAC-supplemented mice, indicating that CoPPIX restored the ability of glucocorticoids to perform negative feedback on the HPA axis.

It is well known that the inhibition of stressor-evoked HPA axis responses at the pituitary level is mediated by the activation of GR (33, 34). Furthermore, NAC reduces the expression of GR in the hypothalamus of mice on a high-cholesterol diet (35) and in the pituitary of healthy rats (13). As expected, we showed that NAC significantly reduced GR protein expression in healthy mice’s pituitary. Treatment with CoPPIX significantly increased the density of GR in this gland of mice supplemented with NAC, strongly confirming that the downregulation of the Nrf2-HO-1 pathway has a causal relationship with the failure of the negative feedback of the HPA axis observed in mice supplemented with NAC. Among the isoforms of GR produced by alternative splicing GRα and GRβ stand out. Unlike GRα, which is the classic receptor responsible for glucocorticoid actions, GRβ cannot bind to glucocorticoids. Nevertheless, GRβ forms a heterodimer with GRα and exerts a dominant-negative effect on GRα-mediated transcription (36–38). Although Otto et al. described that GRβ is not conserved across species and its physiological significance in humans appears questionable, they only evaluated the exons 7, 8, and 9 of the GR loci (39). Currently, it is well known that the mGRβ isoform arises from a distinct alternative splicing mechanism utilizing intron 8, rather than exon 9 as in humans, and that this isoform showed the same properties reported for human GRβ (40, 41). Interestingly, dietary supplementation with NAC significantly reduced the expression of GRα mRNA and increased the expression of GRβ mRNA in the pituitary gland of healthy mice. This data can explain the exact mechanism by which supplementation with NAC completely impairs the HPA axis’ negative feedback, even with a slight but significant reduction in the expression of total GR protein levels. Treatment with CoPPIX increased the expression of GRα mRNA in the pituitary gland of NAC-supplemented mice, however, strongly decreased the expression of GRβ mRNA. Our data showed that the activation of the Nrf2-HO-1 pathway restores the negative feedback of the HPA axis of mice supplemented with NAC by upregulating the expression of the receptor responsible for the glucocorticoid actions, together with a downregulation of the expression of the receptor responsible by the impairment of GRα-mediated activities. In addition, for the first time, we showed that the Nrf2-HO-1 pathway can regulate direct or indirectly the expression of GR isoforms, therefore, our findings contribute novel insights into GR potential regulatory mechanism. Nevertheless, further studies will be required to delineate the exact intracellular pathways involved with the Nrf2/HO-1 pathway-induced glucocorticoid receptor gene expression modulation. Although we strongly believe that activation of the Nrf2-HO-1 pathway in mice supplemented with NAC restores HPA axis negative feedback by modulating the expression of GR isoforms in the mouse pituitary, it is imperative to emphasize that an important limitation of the work is the lack of circulating ACTH levels measurement. We can mainly rule out that NAC supplementation might reduce corticosterone metabolism in the liver and intestine (42), and that CoPPIX treatment might be preventing this possible extra HPA axis effect of NAC supplementation.

We also evaluated the effect of the discontinuation of the supplementation of NAC on the plasma corticosterone levels of mice to evaluate whether NAC-induced downregulation of the HPA axis negative feedback is part of an adaptation period or if this can have a long-lasting effect on the HPA axis stress response. We showed that even after we stopped the dietary supplementation of mice with NAC for 15 days, they showed the same magnitude of increase in circulating corticosterone levels when compared to mice who did not have their supplementation interrupted. Therefore, we can hypothesize that the supplementation of NAC in mice for only 5 days induces a sustainable alteration of the control of glucocorticoid production for at least 15 days. One limitation of our study was that we used only male mice to investigate the effect of NAC on the hyperactivity of the HPA axis in healthy animals, and the effects of NAC on HPA axis function in females should also be considered.

The CoPPIX enhances cellular antioxidant defenses and reduces the production of ROS by inducing the HO-1 signaling pathway, protecting cells from oxidative damage (43). Despite the promising preclinical data, the clinical development of CoPPIX as an antioxidant therapy is in its early stages. Further detailed preclinical and clinical studies are necessary to fully understand the therapeutic potential and safety use of CoPPIX in humans. Moreover, the exploration of CoPPIX and other Nrf2-HO-1 activators holds the potential for developing novel therapeutic strategies for oxidative stress-related diseases (44, 45).

In summary, our results indicate that NAC-induced hyperactivity of the HPA axis in mice is related to the reduction of the activity of the Nrf2-HO-1 pathway in the pituitary gland. This effect seems to be caused by a reduced GRα mRNA and an increased GRβ mRNA transcription, which could lead to a subsequent impairment of the negative feedback of the HPA axis. In addition, the activation of the Nrf2-HO-1 pathway with CoPPIX normalizes the dysregulation in the pituitary gland induced by NAC supplementation and, consequently, stabilizes the negative feedback of the HPA axis after dexamethasone suppression test.

## Data Availability

The original contributions presented in the study are included in the article/**Supplementary Material**. Further inquiries can be directed to the corresponding author.
