# Supplementary material for: Activation of the Nrf2/HO-1 pathway restores N-acetylcysteine-induced impairment of the hypothalamus-pituitary-adrenal axis negative feedback by up-regulating GRα expression and down-regulating GRβ expression into pituitary glands
Source: Front Endocrinol (Lausanne). 2025 Jan 27;16:1500630. doi: 10.3389/fendo.2025.1500630 (PMC11827418; doi:10.3389/fendo.2025.1500630)
Supplement: Supplementary file 1 [file DataSheet1.docx]

**Activation of the Nrf2/HO-1 pathway restores N-acetylcysteine-induced impairment of the hypothalamus-pituitary-adrenal axis negative feedback by up-regulating GRα expression and down-regulating GRβ expression into pituitary glands**

Amanda S. Chaves^1^, Raíssa Duarte Ventura^1^, Maria Florencia Pacini^2^, Nathalia S. Magalhães^1^, Patrícia M. R. e Silva^1^, Marco A. Martins^1^, Ana Rosa Perez^2^, and Vinicius F. Carvalho^1,2^*.

^1^ Laboratory of Inflammation, Center for Research, Innovation, and Surveillance in Covid-19 and Health Emergencies, Oswaldo Cruz Institute, Oswaldo Cruz Foundation, Rio de Janeiro, Brazil.

^2^ Institute of Clinical and Experimental Immunology (IDICER-CONICET UNR), Rosario, Argentina.

^3^ National Institute of Science and Technology on Neuroimmunomodulation (INCT-NIM), Oswaldo Cruz Institute, Oswaldo Cruz Foundation, Rio de Janeiro, Brazil.

^4^ Rio de Janeiro Research Network on Neuroinflammation (RENEURIN), Oswaldo Cruz Institute, Oswaldo Cruz Foundation, Rio de Janeiro, Brazil.

^5^ INOVA-IOC Network on Neuroimmunomodulation, Oswaldo Cruz Institute (IOC), Oswaldo Cruz Foundation, Rio de Janeiro, Brazil.

Supplementary Material

# Supplementary Figures

## Supplementary Figures

**Results**


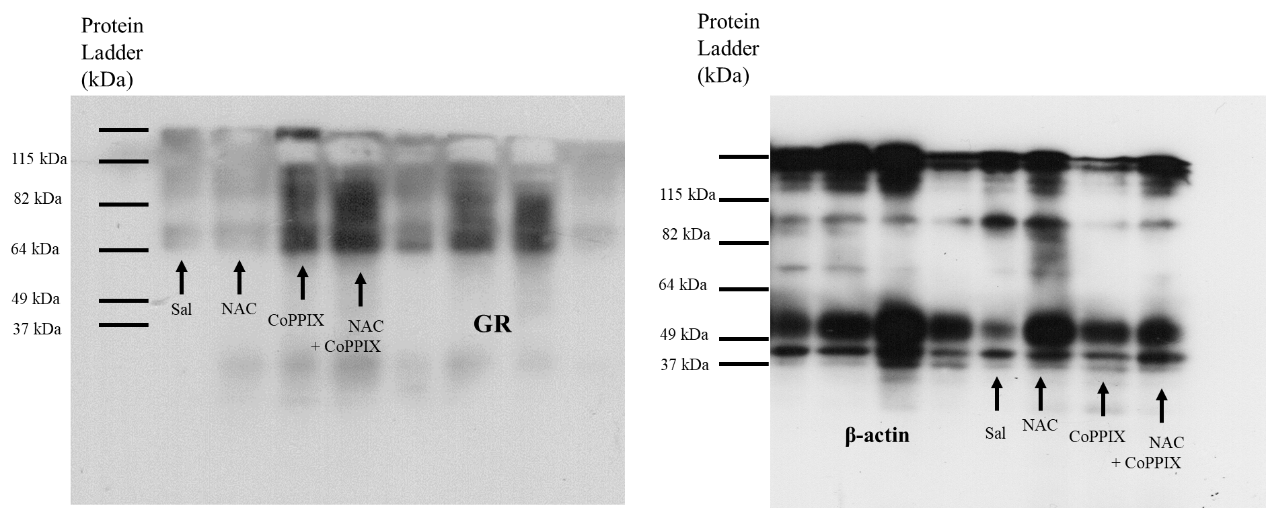


A

B

**Figure S1:** **Western blot evaluation of GR expression in pituitary glands of healthy mice treated with NAC.** Representative full-length blots of GR **(A),** and β-actin **(B)**. Analysis of GR and β-actin expression was performed by western blot. The producers and experiments validated all antibodies. Sal = Saline; NAC = N-Acetylcysteine; CoPPIX; and NAC + CoPPIX.

**Table S1| Characteristics of antibodies used in the study.**

| Antibodies | Dilution Range | Trademark | Application | MW | Isotype |
| --- | --- | --- | --- | --- | --- |
| Anti-gr (G-5):sc-393232 | 1:200 | Santa Cruz | WB | 95/90 kDa | IgG |
| Anti-beta actin (D6A8) | 1:1000 | Cell Signaling | WB | 42 kDa | IgG |
| Anti-rabbit H+L (31460) | 1:10.000 | Invitrogen ThermoFisher | WB | - | IgG |
| Anti-mouse HRP (HAF007) | 1:1000 | R&D | WB | - | IgG |
